# Observation of Exchange Anisotropy in Single-Phase Layer-Structured Oxides with Long Periods

**DOI:** 10.1038/srep15261

**Published:** 2015-10-21

**Authors:** Yan Huang, Guopeng Wang, Shujie Sun, Jianlin Wang, Ranran Peng, Yue Lin, Xiaofang Zhai, Zhengping Fu, Yalin Lu

**Affiliations:** 1 CAS Key Laboratory of Materials for Energy Conversion, Department of Materials Science and Engineering, University of Science and Technology of China, Hefei 230026, P. R. China; 2 Hefei National Laboratory for Physical Sciences at Microscale, University of Science and Technology of China, Hefei 230026, P. R. China; 3 Synergy Innovation Center of Quantum Information & Quantum Physics, University of Science and Technology of China, Hefei 230026, P. R. China; 4 National Synchrotron Radiation Laboratory, University of Science and Technology of China, Hefei 230026, P. R. China; 5 Laser Optics Research Center, US Air force Academy, Colorado 80840, USA

## Abstract

A remarkable exchange bias effect arising from the temperature-dependent interaction among the ferromagnetic-like cluster glasses and antiferromagnetic regions was observed in a newly developed single-phase multiferroic compound of Bi_10_Fe_6_Ti_3_O_30_ which has a nine-layer Aurivillius structure. Inhomogeneous distribution of magnetic Fe ions inside this long-period layered structure was experimentally identified via the atomic level imaging. The results confirmed the presence of the short-range magnetic ordering (the cluster glassy state) and the canted antiferromagnetism, and then the direct interaction among them was further confirmed. Finding of this new single-phase material accompanying this remarkable exchange bias effect would be beneficial to both basic physics understanding and the potential device development.

Exchange anisotropy, usually describing an exchange coupling across an interface between two magnetic materials, plays a crucial role in developing fundamental physics as well as practical applications of the spintronics^1^,^2^,^3^. Spin valve effect^4^ and magnetic tunnel junction^5^, based on the exchange anisotropy effect, have successfully made the giant magnetoresistance (GMR) materials applicable to actual devices and have created a huge economic potential for the area with many anticipated benefits. The most obvious evidence of the exchange anisotropy is the direct measurement of the exchange bias (EB), which manifests itself as a shift of the magnetization hysteresis loop along the magnetic field axis when the system cooling down fully through its Neel temperature under an external magnetic field. EB effect was usually observed in artificially made material systems consisting of ferromagnetic (FM) and antiferromagnetic (AFM) components, for example, FM/AFM bilayers^6^, FM/AFM superlattices^7^, or inhomogeneous spin glasses with microscale FM/AFM domains^8^. Recently, observation of the EB effect has been extended into a few quite new artificial multiferroic heterostructures^9^,^10^, e.g. BiFeO_3_/CoFe^11^ and SrMnO_3_/SrRuO_3_^12^. In such materials, the EB effect could be controlled by an electric field applied to the multiferroic phases on account of the effective magnetoelectric (ME) coupling. Even though the important progresses in this area were achieved in such artificial multiferroic EB materials in recent years, major challenges are still mostly associated with the used artificial structures with different material phases. For example, mismatching of the sintering temperatures for the two used material components will result in chaotic ionic diffusion at the interface, which will then highly restrict the realization of an efficient ME coupling. In addition, coupling between stress and strain arising from the piezoelectric effect of ferroelectric (FE) phase and from the magnetostriction effect of the FM phase could cause an unwanted energy loss. Interestingly, EB effect was recently observed as an intrinsic property in some hole-doped perovskite oxides, such as manganite and cobaltite, due to the inside occurred phase separation leading to the EB across the FM/AFM interfaces^13^,^14^. This research can be considered as an early stage effort to pursue single-phase EB materials, instead of the above discussed artificial structures. Unfortunately, till today single-phase EB materials are still very rare and the observed single-phase EB behaviors were still limited by the small exchange-bias field and the low reachable operation temperatures. Therefore, new single-phase EB materials, with the potential pointing to higher operation temperatures, remain very elusive to the research community.

Layer structured Bi-containing Aurivillius oxides, a material class consisting of alternatively stacked fluorite-like (Bi_2_O_2_)^2+^ units and perovskites-like [A_m−1_B_m_O_3m+1_]^2−^ blocks, have attracted much attention for the coexistence of FE and FM behaviors, with the potential well above the room temperature (RT)^15^,^16^,^17^. Their non-centrosymmetric structure^18^ and the inhomogeneous distribution of Fe/Ti ions inside the octahedral sites accompanying with their strong interactions via oxygen ions appear to play an important role in determining their fascinating coexisted or coupled FE and FM properties^19^,^20^. In this multiferroic material family, for those short-period compounds with the paramagnetic (PM) ground state at the RT, e.g. Bi_5_FeTi_3_O_15_ (*m* = 4), Bi_6_Fe_2_Ti_3_O_15_ (*m* = 5) and Bi_7_Fe_3_Ti_3_O_15_ (*m* = 6), much enhanced FM behaviors can be observed by a newly developed co-doping strategy of substituting cobalt into iron sites^21^,^22^. While compounds of Bi_8_Fe_4_Ti_3_O_24_ (*m* = 7) and Bi_9_Fe_5_Ti_3_O_27_ (*m* = 8), which are with long periods, are mostly AFM materials with a weak ferromagnetism at the RT^20^. Appearance of this weak ferromagnetism in such long-period oxides could trigger the formation of glassy magnetic states due to the coexisted FM and AFM interactions. Therefore, EB could also be expected in such single-phase yet with long-period layered oxides, and thus potentially, could become a new class of single-phase EB materials with the improved operation temperatures. However, very few investigations on studying the EB effect in such long-period materials have been reported so far.

In this work, a distinct exchange bias were observed inside the long-period Bi_10_Fe_6_Ti_3_O_30_ (*m* = 9, B_10_FTO) single-phase ceramics. Interactions between the cluster glasses (CG) and the antiferromagnetic regions in this single-phase layer-structured multiferroic oxide gave rise to a noticeable EB property, of which the remarkable exchange-bias field is larger than those of previously investigated quasi single-phase manganites, cobaltites and of some multiferroic heterostructures^14^,^23^. Structural analyses disclosed that the intrinsic structural disordering is most probably the microcosmic origin of the observe FM and FE performances inside this new complex oxide.

## Results

### Structural characterization

**Figure 1:**
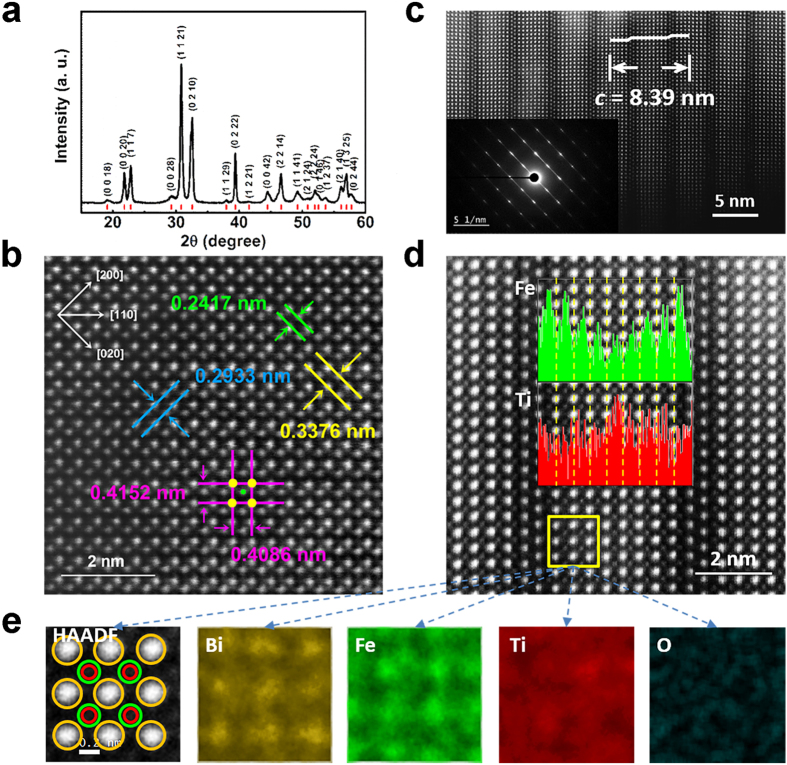
(**a**) Powder XRD pattern of the B_10_FTO sample. The red lines represent the calculated peak positions for odd layered Aurivillius oxides with the space group *B2cb*. (**b**) HAADF image along the [110] zone axis. Yellow and green points are Bi and Fe/Ti atoms, respectively. (**c**) HAADF image and SAED pattern (inset) in (001) section. (**d**) Magnified HAADF image with the lattice period in (001) direction and the EELS analysis (inset) of the elements of Fe (in green) and Ti (in red). (**e**) EDS mapping of the elemental distribution in the selected area marked in (**d**).

X-ray diffraction (XRD) pattern of the B_10_FTO sample is shown in Fig. 1a. All peaks can be well indexed to our calculated peaks using the typical space group *B2cb* for odd layered Aurivillius oxides, and no impurity phases are detected under the current detection accuracy^17^,^19^. Figure 1b shows the high-angle annular dark-filed (HAADF) scanning transmission electron microscope (STEM) image along the [001] zone axis. Lattice parameters were measured to be *a* = 0.5866 nm (=0.2933 nm × 2) and *b* = 0.5793 nm (=0.2417 nm + 0.3376 nm). It distinctly captures that Fe/Ti (green) atoms shift off the geometric centre of the neighboured four Bi atoms (yellow) only along one corner direction. This results in a slight orthorhombic distortion (*b* < *a*), and provides a direct evidence for the ion-displacement driven ferroelectricity of the compound^18^. In addition, the Fe-O-Fe chain, theoretically to be of AFM, may not subtend an angle of 180° because of the tilt in the octahedron, which leads to the canted spin arrangement, giving rise to the weak ferromagnetism via the antisymmetric Dzyaloshinskii-Moriya (DM) interaction^24^.

Characteristic Aurivillius structure with the homogenous nine layered structure of the sample can be evidenced by the HAADF image and the selected area electric diffraction (SAED) pattern of the (001) section, as shown in Fig. 1c and the inset. Lattice parameter *c* was measured to be about 8.39 nm. Magnified HAADF image of the (001) section evidently demonstrates the orderly arrangements of Bi (brighter) and Fe/Ti (grey) atoms, as shown in Fig. 1d, of which eight layers of Bi atoms in the perovskite-like block are sandwiched by two Bi layers in (Bi_2_O_2_)^2−^. Individual columns of Fe/Ti atoms are well packed at the pseudo cubic centre of the adjacent eight Bi atoms except of those in (Bi_2_O_2_)^2−^ layers. It can be clearly observed that the bond length of the two adjacent Bi atomic layers at the outer sides of the perovskite-like block is a little longer than those in the inside block. Detailed distributions of Fe and Ti ions in the lattice structure analysed by the electron energy-loss spectroscopy (EELS) are shown in Fig. 1d inset. It is noted that Fe ions prefers to occupy the outer sites of the sandwiched perovskite layers, while Ti ions prefer the central sites, suggesting an inhomogeneous distribution of B-site cations in the perovskite blocks. X-ray energy dispersive spectroscopy (EDS) mapping images of a selected square area (yellow) in Fig. 1d are shown in Fig. 1e. Bi and Fe atoms can be clearly detected, while the Ti atoms are vague. Less occupation ratio of Ti ions in the selected area may account for this phenomenon, which is quite identical to the EELS results. It can be expected that the inhomogeneous distribution of Fe ions in B sites helps to bring forth a short-range magnetic ordering from the Fe-rich areas, and therefore, some intriguing special magnetic properties.

### Magnetic property

**Figure 2:**
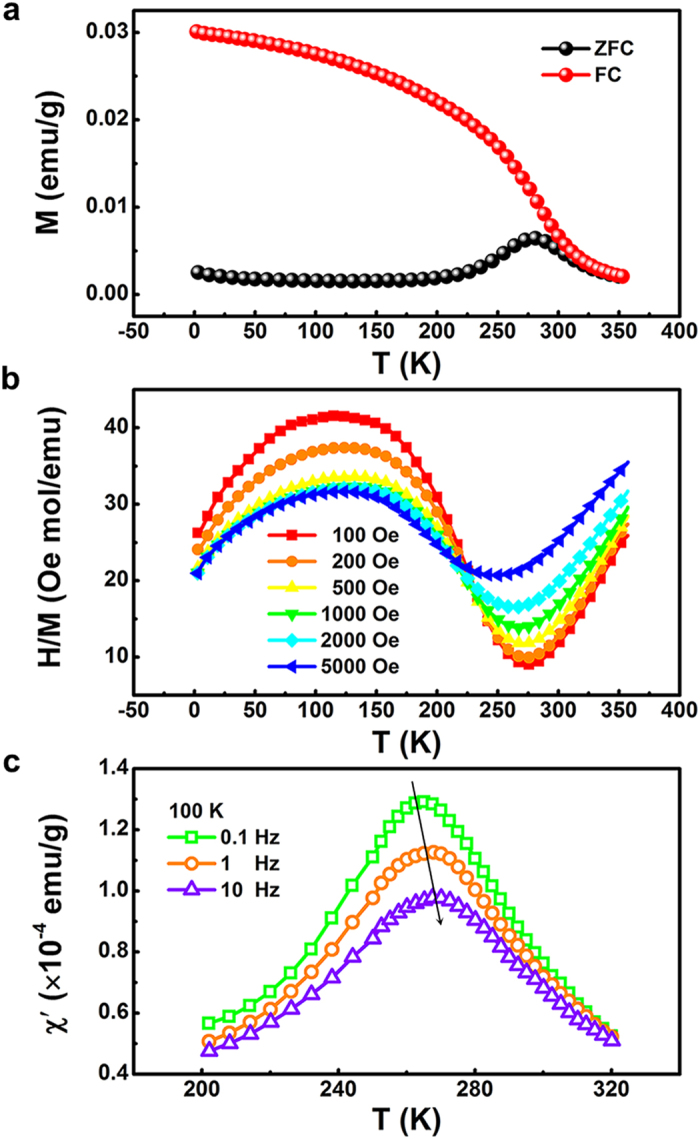
For Bi_10_FTO sample, temperature dependence of (a) magnetization with ZFC and FC processes in an applied field of 200 Oe from 2 K to 350 K, and (b) inverse dc susceptibility in different fields under ZFC mode; (c) real part of ac susceptibility measured under different frequencies at an ac magnetic field of 3.8 Oe.

Temperature dependent magnetization of the B_10_FTO sample was investigated. Figure 2a illustrates the thermomagnetic irreversibility below 300 K. Both zero-field-cooling (ZFC) and field-cooling (FC) curves diverge strongly when lowering the temperature from 360 K to 2 K, implying the onset of some magnetic ordering (or magnetic domain re-orientation) inside the sample. The FC curve below the bifurcation temperature exhibits a “Brillouin-like” behavior, while the ZFC curve shows a broad peak near 270 K. The peak temperature in the ZFC magnetization, defined as *T*_f_, indicates the temperature below which the magnetic moments begin to collectively freeze. *T*_f_ can be also indicated at the valley of the temperature dependence of dc susceptibility (*H*/*M*) under the ZFC mode, as shown in Fig. 2b. It should be noted that both the dc susceptibility and the *T*_f_ change with the applied field. When the applied magnetic field increases, the dc susceptibility reduces and the *T*_f_ moves to the lower temperature, suggesting the presence of some glass-like states. Figure 2c exhibits the real part of the ac susceptibility (*χ′* ) measured at 100 K under different frequencies at an ac magnetic field of 3.8 Oe. *χ′* shows a pronounced peak at around 270 K, which shifts slightly to higher temperatures with lowering intensity at higher driving frequencies. Furthermore, these curves measured at different driving frequencies overlap at 320 K. Such phenomena confirm the glassy nature of the materials in which the competing magnetic interactions are present below a characteristic temperature^25^. The large irreversibility of ZFC-FC curves in the dc magnetization and the frequency dependent anomaly in *χ′* indeed confirm a short-range magnetic ordering, which may associate with a CG phase’s existence, identical to our above conjecture on the structural modulation on B_10_FTO’s magnetic properties.

**Figure 3:**
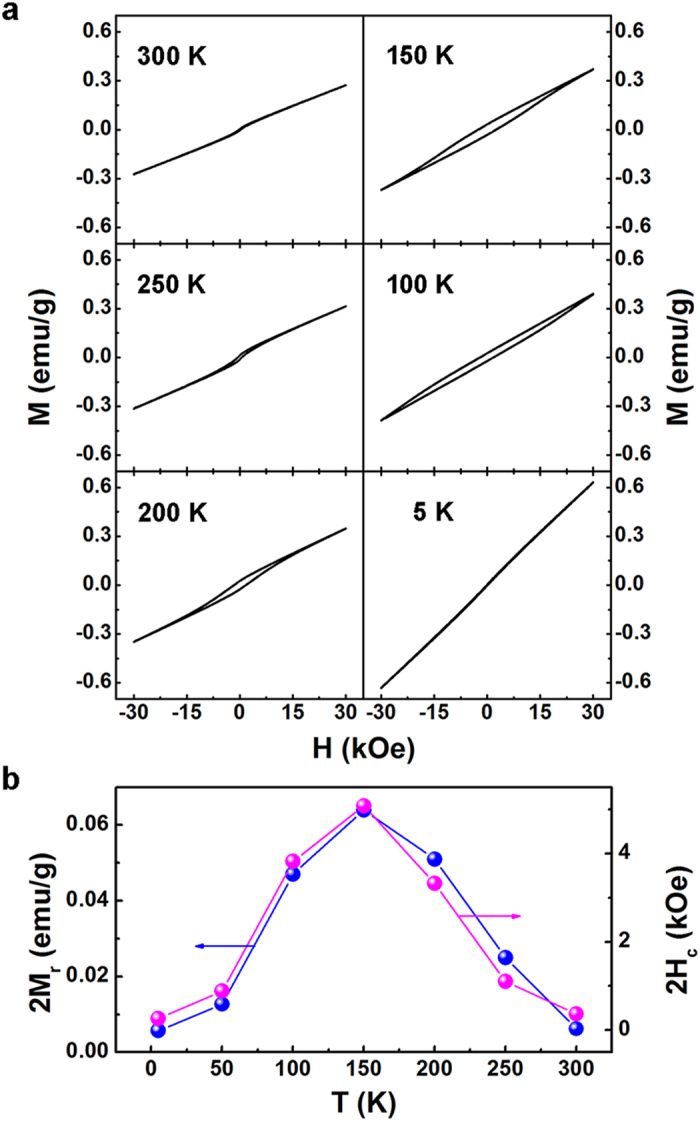
(**a**) Magnetic isotherms of the B_10_FTO sample measured between 300 K and 5 K. (**b**) Remanent magnetization (*M*_*r*_) and coercive fields (*H*_*c*_) as a function of temperature.

To get more details about the magnetic properties, magnetic isotherms of the sample measured between 5 K and 300 K are shown in Fig. 3a. When the temperature is 300 K, the *M*-*H* plot exhibits a small hysteresis unsaturated up to a large magnetic field of ±3 T, indicating a superposition of both FM and AFM components. When the temperature reduces to 250 K and 200 K, the hysteresis loops become more obvious. When the temperature continuously cooling down to 150 K and then to 100 K, the curves split up from the line with an enlarged remanent magnetization (*M*_r_) and coercive field (*H*_c_). At 5 K, the plots with the performance of an almost linear behaviour, steeply rising with the applied magnetic field, reveal an AFM characteristic. From the *M*(*H*) dependencies, *M*_r_ and *H*_c_ were determined as a function of measured temperatures and the results are illustrated in Fig. 3b. Both *M*_r_ and *H*_c_ values increase first and then reduce with a further decrease of temperature. The largely enhanced coercivity is usually observed in FM/AFM bilayers, as the spin fluctuations in the AFM layer may generate an additional anisotropy in the FM layer via an interaction^26^.

### Exchange bias

**Figure 4:**
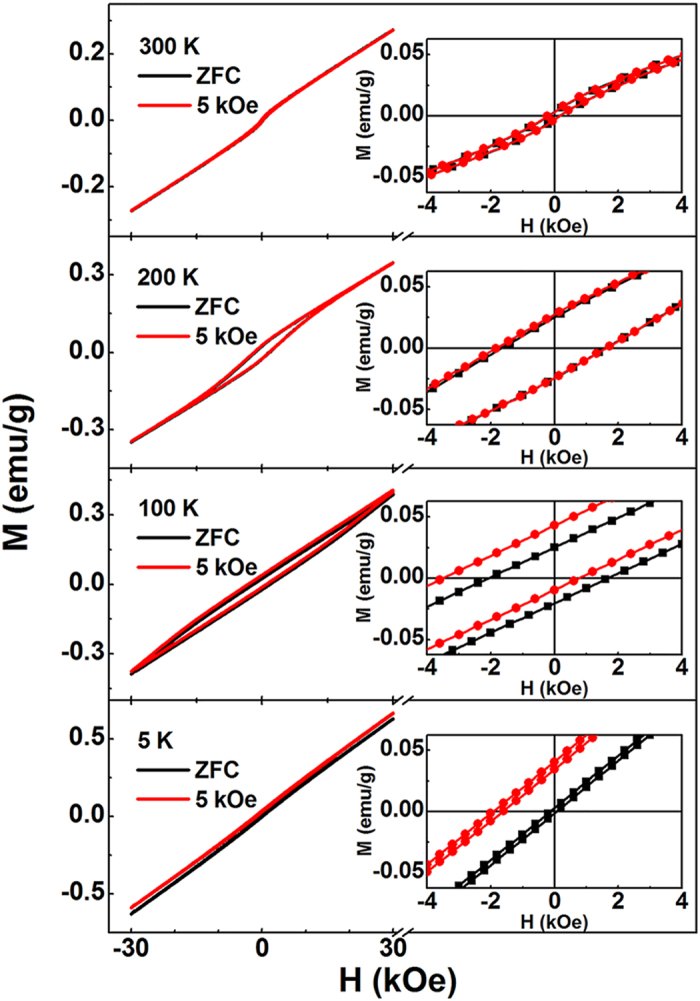
Hysteresis loops measured in ZFC and FC modes at different temperatures. Insets show the enlarged view of the low field region.

*M*-*H* curves of the B_10_FTO sample cooling from 360 K down to various temperatures at the magnetic field of 5 KOe are shown in Fig. 4, and compared with those cooling in the ZFC process. The curves in two cooling processes are almost identical at 300 K. Nevertheless, when the temperature decreases to 200 K, the FC curve begins to shift along both the up *M* axis and the right *H* axis, suggesting an EB effect. When the temperature continuously cools to 100 K, the displacement of the FC loop becomes much more evident with an exchange-bias field *H*_E_ = 1468 Oe, which is much larger than that of La_0.82_Sr_0.18_CoO_3_ (*H*_E_ = 72 Oe) measured at 5 K in the cooling field of 10 KOe^14^, and even larger than that of multiferroic epitaxial heterostructure Py/YMnO_3_/Pt (about 60 Oe) measured at 2 K after field cooling at 3 KOe^23^. As reported before, the hysteresis loop, for a specific FM/AFM case where the AFM has a parasitic FM, would shift only along the *M* axis but not the *H* axis^27^. In fact, in our case this shift moves along both axes, and this indicates that the observation of the EB phenomenon here can not only be explained by the AFM/parasitic FM combination, but also by the above discussed temperature-dependence CG state’s existence. When the temperature is slightly below *T*_f_, the FM-like CG may partially line up with the applied field, while the neighbouring AFM spins remain random; when the temperature becomes further lower (e. g. 200 K), the AFM spins next to the CG may arrange along certain directions due to the exchange interaction at the CG/AFM interfaces; when the temperature is far below the *T*_f_ (e. g. 100 K), the AFM spins at the interfaces may exert a microscopic torque to the CG domains preventing them from turning back to the original orientation when the magnetic field is removed. The above speculation can be verified by the observation that FC hysteresis loops shift both toward the negative field as well as toward the positive magnetization^8^. Further displacement of the loop at 5 K may result from the enhanced magnetic irreversibility induced by the enlarged frozen CG regions as well as the reduction of the coercive field.

**Figure 5:**
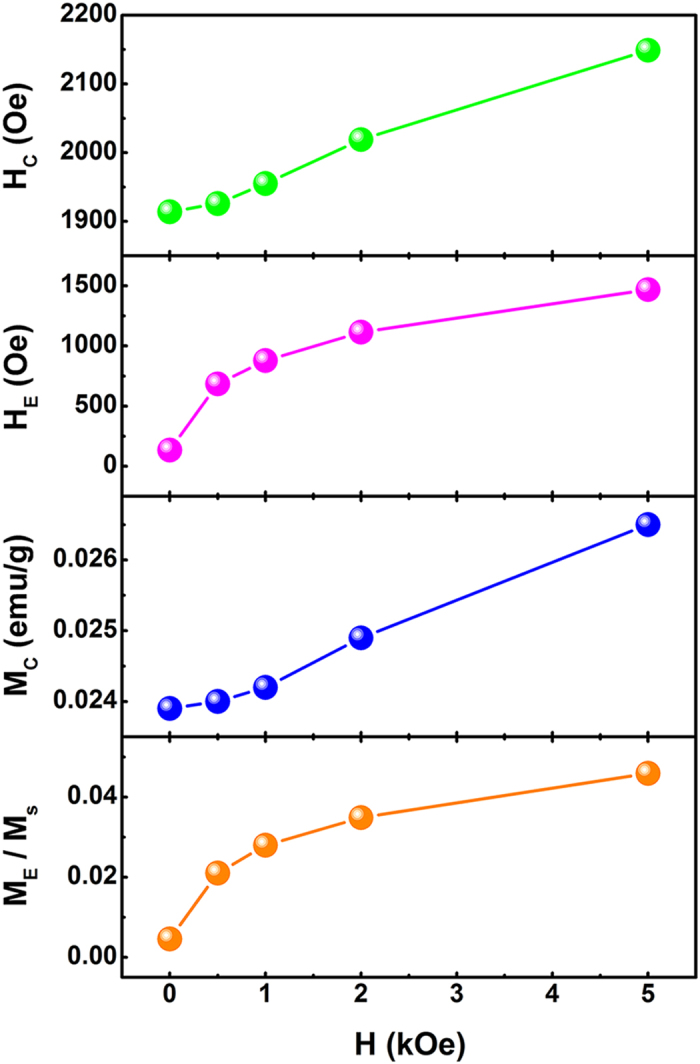
Cooling field dependence of *H*_*C*_, *H*_*E*_, *M*_*C*_ and *M*_*E*_*/M*_*S*_ of the B_10_FTO sample measured at 100 K.

Considering that the CG phase can be influenced by the applied cooling field (*H*_cool_), the function of *H*_cool_ on the exchange-bias parameters at 100 K was shown in Fig. 5. The magnetic coercivity, *M*_C_, and remanence asymmetry, *M*_E_, show similar tendency with the coercive field *H*_C_ and exchange-bias field *H*_E_, respectively. All the parameters mentioned above increase with the enhanced *H*_*cool*_ though their rates differ. At high *H*_*cool*_, *H*_*E*_ and *M*_*E*_/*M*_*S*_ (*M*_*S*_ is the saturate magnetization) tend to be saturate but *H*_*C*_ and *M*_*C*_ still sharply increase. This phenomenon is qualitatively analogous to that of FM/AFM thin films, of which *H*_*cool*_ can be described as the function of *H*_*E*_ with the following relation^13^:


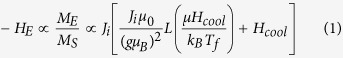


where *g* is the gyromagnetic factor, *J*_*i*_ represents the surface exchange constant, *L*(*x*) is the Langevin function and *k*_*B*_ denotes Boltzmann’s constant. *μ*_*B*_ = *N*_*ν*_*μ*_*0*_, here *μ*_*B*_ and *μ*_*0*_ are the Bohr magneton and the magnetic moment of the Fe core spin, respectively, and *N*_*ν*_ is the number of spins inside the AFM volume. As shown in equation (1), the competition between the exchange interaction and the cooling field plays an important role. For small *H*_*cool*_, the first term usually dominates, and therefore, *H*_*E*_ (<0) and *M*_*E*_/*M*_*S*_ depends on *J*_*i*_^2^, which increase rapidly at first. While for large *H*_*cool*_, the proportion of second term increases and cannot be ignored, and thus, the increasing rate of the absolute value of *H*_*E*_ and *M*_*E*_/*M*_*S*_ should reduce.

**Figure 6:**
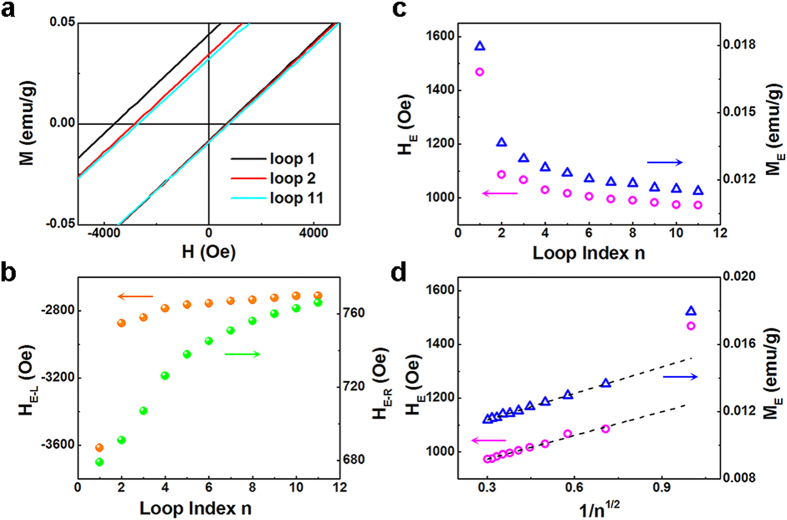
(**a**) The first, the second and the eleventh loops measured at 100 K after cooling in 5 KOe from room temperature. (**b**) The field cycles dependence ofright and left field branches of exchange-bias field. (**c**) The field cycles dependence of *H*_*E*_ and *M*_*E*_ measured at 100 K cooling in 5 KOe from room temperature, and (**d**) the linear fits of *H*_*E*_ and *M*_*E*_ for *n* > 1.

Another characteristic in exchange-bias systems is the training effect, which describes the reduction of the exchange-bias field *H*_E_ when cycling the system through several consecutive hysteresis loops. The consecutive hysteresis loops were measured at 100 K after the field cooling in 5 KOe, as shown in Fig. 6a. Compared with the right branch (*H*_*E-R*_), the shift of left branch (*H*_*E-L*_) is much more obvious, as shown in Fig. 6b. For the first cycle, *H*_*E-L*_ rises faster than right branch, however, for the later cycles, *H*_*E-L*_ saturates and *H*_*E-R*_ keeps on increasing. The fact that field cycling seems to affect *H*_*E-L*_ more strongly than *H*_*E-R*_ might suggest the thermal activation in the CG being dominant in the field-training response^28^. Domains in the CG/AFM regions may get switched due to thermal activation for each branch of the loop. The number of such reversals ought to be the same on either branch because it is only sensitive to the FM magnetization with the same magnitude in both directions of the field sweep. The FM-like CG, which is not strongly biased, experiences a different magnitude of field during the forward and reverse branches, since the loop is offset from *H* = 0. The number of thermally activated reversals in this set of weakly biased region will be greater for the reverse branch of loop where the field magnitude is larger. Any associated changes in the CG/AFM regions would therefore be larger near *H*_*E-L*_ compared to *H*_*E-R*_.

The relaxation of the remanence asymmetry is evident, and both the exchange-bias field (*H*_*E*_) and the magnetization shift (*M*_*E*_) reduce with magnetic field cycling as shown in Fig. 6c. The usual experimentally observed relationship between *H*_*E*_ (*M*_*E*_) and *n* is given by


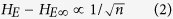


Where *H*_*E∞*_ is the exchange-bias field in the limit of infinite loops. Figure 6d shows the best fits with this empirical relation to both *H*_*E*_ and *M*_*E*_ which is in satisfactory agreement with the experimental data for *n* > 1. The fitting parameters *H*_*E∞*_ = 885 Oe and *M*_*E∞*_ = 0.0099 emu/g are calculated from the fitted curves. The data point at *n* = 1 significa*n*tly exceeds the value when extrapolating the fit. Previous investigations have revealed the breakdown of the power-law behavior at *n* = 1. Although the power-law decay of exchange bias has been widely observed, its origin remained unexplained. Recently, the training effect in FM/AFM heterostructures in the framework of nonequilibrium thermodynamics is considered^29^. It was suggested that consecutively cycled hysteresis loops of the FM top layer trigger the spin configurational relaxation of the AFM interface magnetization toward equilibrium and a recursive formula is obtained to describe the *n* dependence of *H*_*E*_ (*M*_*E*_):


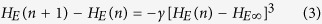


where *γ* is a sample-dependent constant. Using the initial value of *H*_*E*_ (1) = 1468 Oe obtained from experiments, *γ* = 1.683 × 10^–6^ Oe^−2^ and *H*_*E∞*_ = 858 Oe, the theoretical data of *H*_*E*_ are calculated from the implicit sequence (3). Similarly, the theoretical data of *M*_*E*_ are obtained with *M*_*E*_ (1) = 0.01795 emu/g, *γ* = 5095 (emu/g)^−2^ and *M*_*E∞*_ = 0.0085 emu/g. It is found that both theoretical data are well coincident with experimental results not only for *n* > 1 but also for *n* = 1. Thus, the spin configurational relaxation model can describe our experimental results well. It is likely that the consecutive reversion of the CG magnetization triggers the configurational relaxation of the interfacial AFM spins toward equilibrium and results in the training effect.

### Ferroelectric and dielectric properties

**Figure 7:**
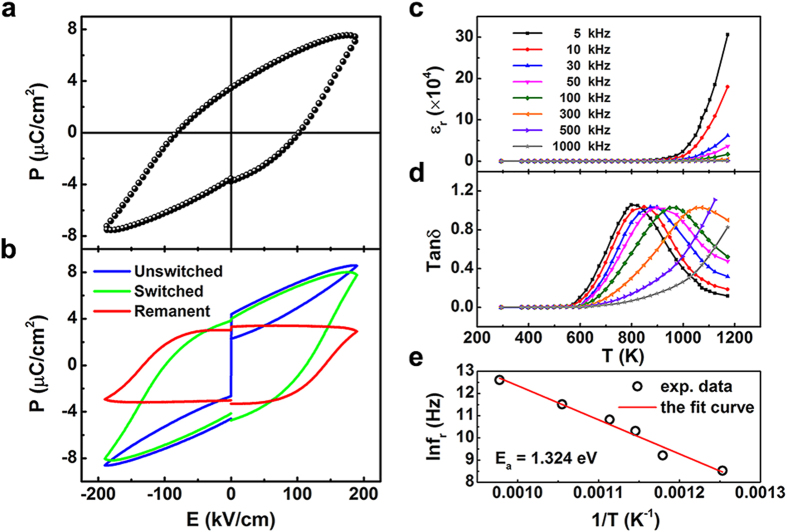
Polarization-electric field hysteresis loop (a) and remanent polarization hysteresis loop (b) under an electric field of 190 KV/cm at RT. Temperature dependence of dielectric constant (**c**) and dielectric loss (**d**) at different frequencies; (**e**) The Arrhenius plot of the relaxation frequency vs. the inverse of the peak temperature.

Figure 7a shows typical ferroelectric hysteresis loops of the Bi_10_FTO ceramic at RT. The value of remnant polarization 2*P*_*r*_ is about 7 *μ*C/cm^2^ under an applied electric field up to 190 KV/cm. To rule out the contribution from the artificial polarization associated with the electrical leakage, the remanent polarization hysteresis loop for the same sample was recorded and is shown in Fig. 7b. The loop is nearly closed and the value of remanent polarization from this loop is 6.35 *μ*C/cm^2^, close to the above 2*P*_*r*_ value, suggesting low artificial polarization contribution. The off-center distortion of the perovskite-like block originating from lone pairs of Bi^3+^ should account for the intrinsic FE of Bi_10_FTO.

Figure 7c,d show the temperature dependences of dielectric constant (ε′) and dielectric loss (tan δ) of Bi_10_FTO measured at various frequencies, respectively. The ε′ increases with rising the testing temperature, however, the peak related to the ferroelectric phase transition is not observed within the range of the testing temperatures, suggesting a high Curie temperature for Bi_10_FTO. The loss peak shifts toward a higher temperature with increasing the testing frequency. Normally, the frequency dependence of the loss peak is deemed as typical characteristics of the relaxation ferroelectrics. Variation of the peak temperature of dielectric loss at different frequencies can be fitted using the Arrhenius law:


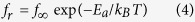


where *f*_*∞*_ is the relaxation frequency at an infinite temperature, *E*_*a*_ the activation energy, and *k*_*B*_ the Boltzmann constant. As shown in Fig. 7e, the relaxation parameter *E*_*a*_ can be estimated as 1.324 eV, which is larger than those for oxygen vacancies migrations in oxide ceramics, e.g., 0.87 eV in Bi_4_Ti_3_O_12_^30^. High activation energy of the sample may be resulted from its structural distortions mainly.

## Discussion

The regular stacking of the (Bi_2_O_2_)^2+^ layers and the perovskite-like blocks and the inhomogeneous distribution of magnetic ions inside the perovskite-like blocks make the Bi_10_Fe_6_Ti_3_O_30_ ceramic, a long-period Aurivillius oxide, present a cluster glass behavior below the room temperatures. In addition, the interaction between the cluster glasses and the antiferromagnetic regions triggers a large exchange bias in Bi_10_Fe_6_Ti_3_O_30_. This observation may open a window for seeking more single–phase compounds with novel exchange bias features.

In summary, distorted structures in a new and single-phase nine-layer Aurivillius compound Bi_10_Fe_6_Ti_3_O_30_ were directly observed. The inhomogeneous distribution of magnetic ions revealed the presence of the short-range magnetic ordering and the canted antiferromagnetism. Exchange bias phenomenon was obviously observed with the displacement of the hysteresis loop as well as the enhancement of coercive field due to the interaction between the CG and AFM regions. Finding of this new single-phase material accompanying a remarkable EB effect would be meaningful to both basic physics understanding and the potential device development.

## Methods

Bi_10_Fe_6_Ti_3_O_30_ (B_10_FTO) powders were synthesized by the modified Pechini method^22^. Ti(C_4_H_9_O)_4_, Bi(NO_3_)_3_·5H_2_O and Fe(NO_3_)_3_·9H_2_O were dissolved into dilute nitric acid solution in a stoichiometric ratio with proper amounts of citric acid (C_6_H_8_O_7_) and ethylene diamine tetraacetic acid (EDTA). The resulted precursor was heated in a beaker until it self-igniting to xerogel powders after the solvent evaporation. The obtained powders were fired at 1023 K for 2 h to remove carbon residues and to form well crystallized structures. The B_10_FTO ceramic was finally prepared by cold-pressing the powder into a disk and then sintered at 1233 K for 3 h in air.

Crystalline structure of the B_10_FTO sample was investigated using an X-ray diffractometer (TTR-III, Rigaku) with the Cu-K_α_ radiation. Atomic structures were observed using an aberration corrected scanning transmission electron microscope (STEM) (JEM-ARM200F, JEOL) equipped with the X-ray energy dispersive spectroscopy (EDS) (X-max80, Oxford Instruments) and an electron energy-loss spectroscopy (EELS) (GIF Quantum 965, Gatan). Dc FM measurements were performed using a physical property measurement system (PPMS) (Dynacool, Quantum Design) and the ac part was recorded using a superconducting quantum interface device (SQUID) (MPMS XL-7, Quantum Design). FE measurements were conducted using a precision LC FE analyzer (Radiant Technology) at the room temperature. Dielectric property was characterized using an impedance analyzer (HP 4294A) in the temperature range of 300 to 1200 K.

## Additional Information

**How to cite this article**: Huang, Y. *et al.* Observation of Exchange Anisotropy in Single-Phase Layer-Structured Oxides with Long Periods. *Sci. Rep.* **5**, 15261; doi: 10.1038/srep15261 (2015).
